# Automatic segmentation of prostate MRI using convolutional neural networks: Investigating the impact of network architecture on the accuracy of volume measurement and MRI-ultrasound registration

**DOI:** 10.1016/j.media.2019.101558

**Published:** 2019-12

**Authors:** Nooshin Ghavami, Yipeng Hu, Eli Gibson, Ester Bonmati, Mark Emberton, Caroline M. Moore, Dean C. Barratt

**Affiliations:** a Centre for Medical Image Computing, Department of Medical Physics and Biomedical Engineering, University College London, London, UK; b Wellcome/EPSRC Centre for Interventional and Surgical Sciences, University College London, London, UK; c Siemens Healthineers, Princeton, USA; d Division of Surgery & Interventional Science, University College London, London, UK

**Keywords:** Medical image segmentation, Neural networks, Prostate cancer, MRI

## Abstract

Convolutional neural networks (CNNs) have recently led to significant advances in automatic segmentations of anatomical structures in medical images, and a wide variety of network architectures are now available to the research community. For applications such as segmentation of the prostate in magnetic resonance images (MRI), the results of the PROMISE12 online algorithm evaluation platform have demonstrated differences between the best-performing segmentation algorithms in terms of numerical accuracy using standard metrics such as the Dice score and boundary distance. These small differences in the segmented regions/boundaries outputted by different algorithms may potentially have an unsubstantial impact on the results of downstream image analysis tasks, such as estimating organ volume and multimodal image registration, which inform clinical decisions. This impact has not been previously investigated. In this work, we quantified the accuracy of six different CNNs in segmenting the prostate in 3D patient T2-weighted MRI scans and compared the accuracy of organ volume estimation and MRI-ultrasound (US) registration errors using the prostate segmentations produced by different networks. Networks were trained and tested using a set of 232 patient MRIs with labels provided by experienced clinicians. A statistically significant difference was found among the Dice scores and boundary distances produced by these networks in a non-parametric analysis of variance (*p* < 0.001 and *p* < 0.001*,* respectively), where the following multiple comparison tests revealed that the statistically significant difference in segmentation errors were caused by at least one tested network. Gland volume errors (GVEs) and target registration errors (TREs) were then estimated using the CNN-generated segmentations. Interestingly, there was no statistical difference found in either GVEs or TREs among different networks, (*p* *=* 0.34 and *p* *=* 0.26*,* respectively). This result provides a real-world example that these networks with different segmentation performances may potentially provide indistinguishably adequate registration accuracies to assist prostate cancer imaging applications. We conclude by recommending that the differences in the accuracy of downstream image analysis tasks that make use of data output by automatic segmentation methods, such as CNNs, within a clinical pipeline should be taken into account when selecting between different network architectures, in addition to reporting the segmentation accuracy.

## Highlights

• Six openly-available neural networks for prostate MR segmentation were compared, based on experiment results using 232-patient clinical imaging and label data.
• Statistically significant difference in segmentation accuracy was found in one network.
• No statistically significant difference was found in subsequent clinical applications, for volume estimation or image registration where these segmentations were used.

## Graphical abstract

**Figure:**
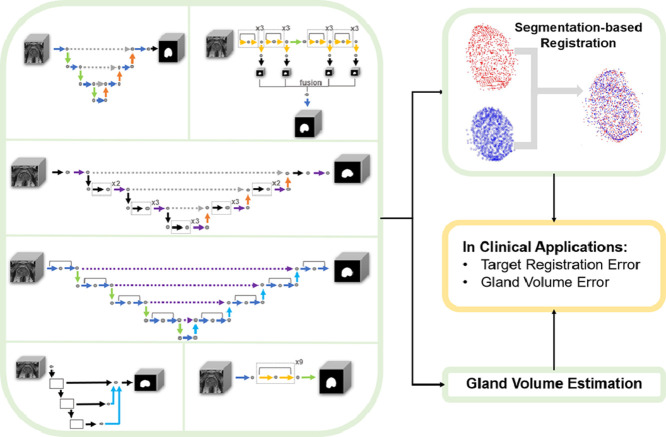

## 1 Introduction

Prostate cancer is the most commonly diagnosed non-cutaneous cancer in men in many parts of the Western world and is a major cause of cancer-related death internationally (Cancer Research UK, 2015). Multi-parametric magnetic resonance imaging (mp-MRI) is emerging as a clinically useful tool for detecting and localising prostate cancer. Results from the recent PROMIS and PRECISION studies, for instance, suggest that mp-MRI may be a valuable triage tool for clinically-significant disease to reduce the number of transrectal biopsies (Ahmed et al., 2017, Kasivisvanathan et al., 2018). In addition, mp-MRI is increasingly being used to target suspicious regions during biopsy and therapy, with or without the aid of a computer-assisted MRI-ultrasound (US) fusion system (Robertson et al., 2013).

**Fig. 1:**
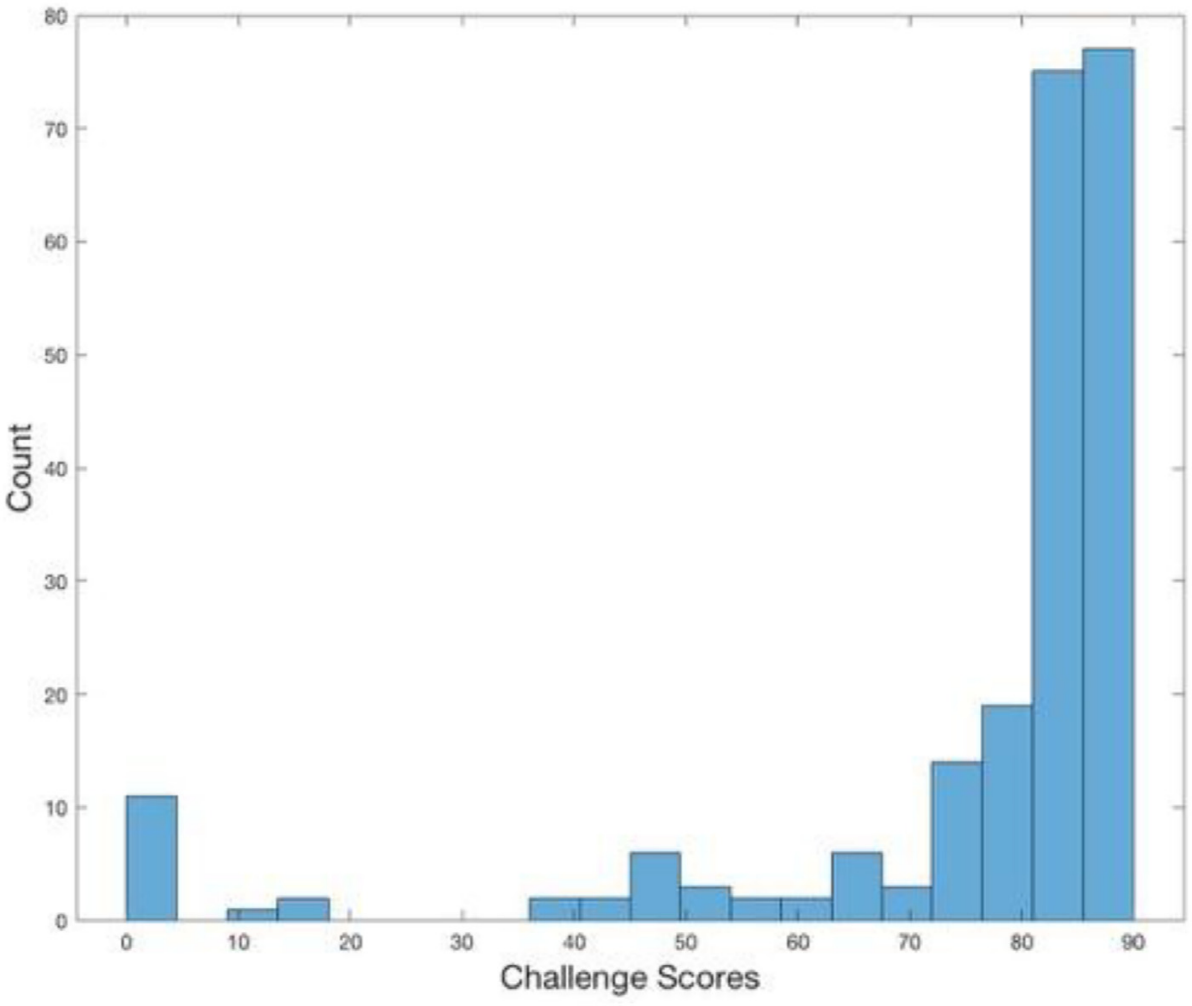
Histogram of the results from the PROMISE12 challenge.

Deep learning methods, especially supervised classification methods based on convolutional neural networks (CNNs), have been successful in the field of medical imaging for segmenting the anatomy of interest (Litjens et al., 2017). For example, these networks have produced higher accuracies for automatic prostate segmentations from T2-weighted MRIs, compared with alternative segmentation approaches (Litjens et al., 2017). An example of these networks include the V-Net (Milletari et al., 2017), which was proposed to segment the prostate gland from T2-weighted MRIs in 2016, and has since been adapted in several different applications (Gibson et al., 2018, Han, 2017, Milletari et al., 2017, Roy et al., 2017). More recently, other variations of CNNs have also been proposed for prostate image segmentation, including (Zhu et al., 2017, Yu et al., 2017, Clark et al., 2017, Tian et al., 2017). At the time of writing, all the top five prostate segmentation algorithms submitted to the PROMISE12 challenge (Litjens et al., 2014; MICCAI Grand Challenges, 2012) adopted CNNs, with the highest performing methods generating average Dice scores and boundary distances of 0.90 and 1.71 mm on whole gland segmentation, respectively. Fig. 1 shows a histogram of the results from the PROMISE12 table, with many submitted algorithms centred around a score of 82–89, where the score is calculated based on the average of multiple accuracy metrics. With all these variations of CNNs for prostate MRI segmentation, a direct quantitative comparison of different CNN architectures on a single large data set, especially those with open-source implementations (not a requirement for submitting to the Challenge) is important, but to date has not been available to our research community.

Partly limited by the test data size of 30 images provided in the PROMISE12 Challenge, the diminishing statistically significant differences among top performing segmentation algorithms (Gibson et al., 2017a) can complicate interpreting these differences, if any, in segmentation accuracy. In other research fields, however, examples of networks that demonstrated statistical differences between different architectures include the introduction of residual networks (He et al., 2016) and densely connected networks (Huang et al., 2017). Both have demonstrated significantly improved results in computer vision tasks, and have been incorporated for medical image segmentation as shown by Ghavami et al. (2018a) and Gibson et al. (2018), respectively.

Perhaps more importantly, assessing the value of adopting different CNN architectures in clinical applications requires evaluating their performance within a pipeline of clinical tasks. However, the vast majority of studies in the literature on prostate MRI segmentation focus on evaluating the accuracy of segmentation techniques in isolation without considering how segmentation errors propagate through subsequent computational tasks within a clinical workflow. Segmentation of the prostate from MRIs is important for several potential clinical tasks. One application includes calculating the gland volume estimation which can be used for measuring drug-induced prostate volume changes (Moore et al., 2017), for correlation with cancer volume (Matsugasumi et al., 2015) and for detecting significant cancer (Thompson et al., 2016, Khalvati et al., 2016). Other applications also include for longitudinal analysis of patients undergoing active surveillance (Stamatakis et al., 2013; Diaz et al., 2015, Ouzzane et al., 2015, Nguyen et al., 2015, Lai et al., 2017), and as part of segmentation-driven multi-modal registration to support MRI-targeted transrectal-ultrasound (TRUS) guided biopsy and therapy (Ouzzane et al., 2015, Narayanan et al., 2009, Onofrey et al., 2017). Relating the accuracy of these clinical measures to the accuracy of the output of different MRI segmentation networks (for example, those submitted to the PROMISE12 Challenge (Fig. 1)), with which they are computed, has not yet been investigated, but clearly has important implications for the selection and deployment of these networks within clinical workflows.

However, comparison of deep-learning-based segmentation algorithms also faces significant challenges such as the requirement of test data size, in addition to the dependency on the hyperparameter selection, including initial learning rate, model size (number of layers and feature channels in each layer) and regularisation methods such as weight decay. Cross-validation for hyperparameter searching is effective in resampling the limited data (a common restriction in medical image computing applications), but is likely to produce “over-optimistic” models due to information bleeding (Valdes and Interian, 2018). Arbitrary hyperparameter selection would lead to less clinically meaningful comparison between merely sub-optimally-designed networks, while marginalising these hyperparameter spaces for architectural comparison is computationally prohibitive and has little practical value. Therefore, we split our data into development and hold-out sets before optimising the hyperparameters using cross-validation on the development set. The details of the experiment design and its implementation for prostate segmentation on MRIs are provided in Section 2.

In this study, our aim was to compare the prostate segmentation accuracy of six different CNN architectures, in terms of two segmentation metrics*,* gland volume estimates and registration errors, the latter two of which are based on the automatic segmentations, and the differences between these errors. This work aims to: 1) demonstrate deviations in segmentation accuracy due to varying network architectures, and 2) to estimate clinically relevant impact that can potentially be caused by these deviations. In turn, the contributions of this work are summarised as follows: 1) A quantitative comparison of six open-source segmentation algorithms is carried out, each one adapted to prostate MRI segmentation, trained using an extensive hyperparameters tuning, and tested on an independent hold-out data set; 2) A comprehensive set of segmentation accuracy results are reported and compared, over these different networks; 3) clinically relevant results pertaining to gland volume estimation and MRI-TRUS image registration, are reported and compared. Investigating the disagreement between the clinically relevant results and the segmentation accuracy is of great importance.

## 2 Methods

### 2.1 Networks for comparison

We chose six network architectures in this study: UNet, VNet, HighRes3dNet, HolisticNet, DenseVNet, and Adapted UNet. Our inclusion criteria included relevance, availability and reproducibility, as the implementations of these six networks are readily accessible and they have been already applied on the same or closely-relevant applications. For example (re-)implementations of the first five are available on the NiftyNet Platform (Gibson et al., 2017b) and the Adapted UNet (Ghavami et al., 2018a) has been developed in our group, with a minimal adaptation to the original 3D UNet. While these open-source development platforms are readily accessible to the research community, it is noteworthy that there are other recently proposed networks such as those based on attention and region proposal mechanisms.

**Fig. 2:**
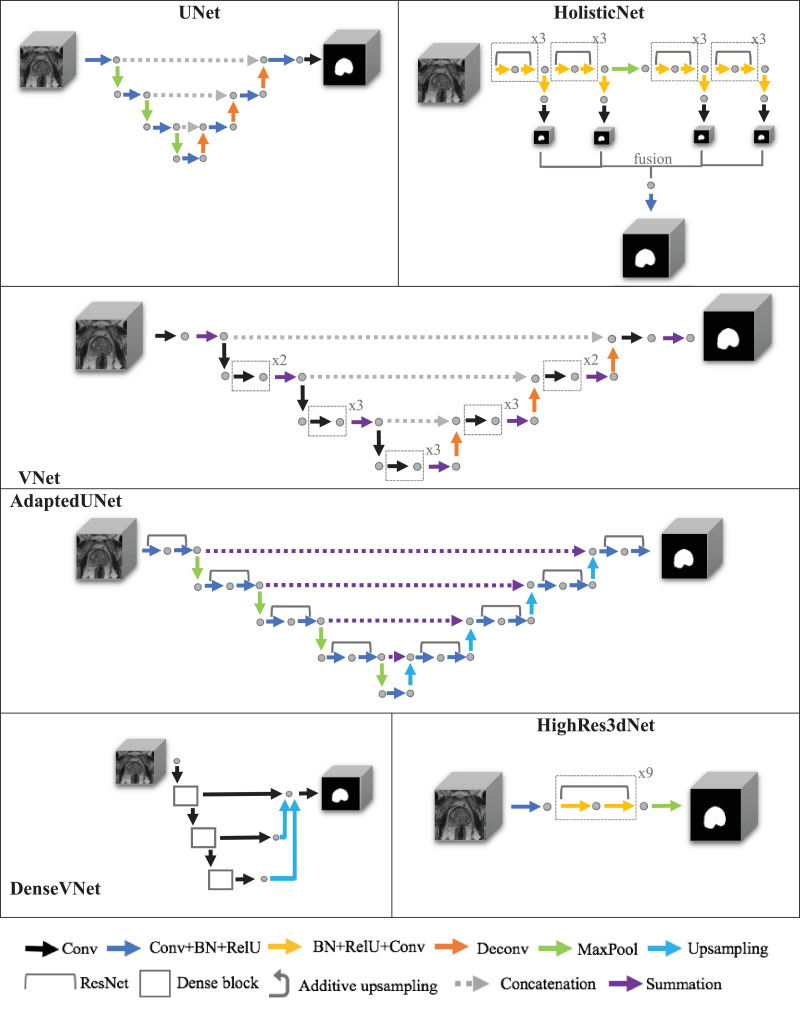
Architecture of the six networks used for this comparison study. Different coloured arrows represent different architecture parts of the networks to visualise similarities and differences between them.

The 3D UNet (Cicek et al., 2016) is one of the earliest proposed 3D fully convolutional neural networks originally proposed for segmenting kidney embryos on xenopus and reported an average intersection over union (IoU) of 0.7 for this application. The VNet (Milletari et al., 2016) also adopted a volumetric CNN architecture, focusing on prostate segmentation from MRI by which, an average Dice score ± std and average Hausdorff distance+std of 0.87 ± 0.03 and 5.71 ± 1.20 mm, respectively, was obtained. VNet was evaluated on the PROMISE12 dataset. HighRes3dNet is an adapted CNN architecture based on dilated convolutions and residual connections (Li et al., 2017), proposed for brain structures, achieving an average Dice score ± std of 0.84 ± 0.02. HolisticNet (Fidon et al., 2017) is inspired by previous holistically-nested edge detection algorithms (Xie and Tu, 2015), which uses a generalisation of the Dice based on Wasserstein distance as the training loss. HolisticNet was proposed for brain tumour segmentation, reporting an average Dice of 0.89. Based on the VNet architecture, DenseVNet (Gibson et al., 2018) was proposed to incorporate the densely-connected feature stacks. Compared to three other state-of-the-art algorithms, statistically significantly higher Dice scores for spleen, stomach, oesophagus, liver, left kidney, gall bladder and pancreas were achieved. Finally, we compare the 3D Adapted UNet based on the original work segmenting prostate gland from 2D TRUS images (Ghavami et al., 2018a, Ghavami et al., 2018b), with an average Dice score ± std and an average boundary distance ± std of 0.91 ± 0.12 and 1.23 ± 1.46 mm, respectively. The original 2D network was extended to 3D by replacing all 2D operations such as convolution and pooling with the respective 3D operations. Table 1 summarises the experiment details of each network used in their original work, while Fig. 2 illustrates their network architecture. The reader is referred to the original papers and published code for other network details, which are kept unchanged in this work, for the interest of brevity.

**Table 1:** Information regarding the networks chosen for this comparison study.

Network	Total data size	Training vs testing data size	Application	Comparison to other methods	Statistical significance Testing applied in comparison?
UNet	3 Xenopus samples-77 Slices (3-fold cross-validation)	51–52 per fold for training	Xenopus kidney embryos	2D UNet	No
		25–26 per fold for testing (77 across all folds)			
VNet^a^	80 subjects (single training-testing-split)	50 (training)	Prostate	Imorphics	No
		30 (testing)		ScrAutoProstate	
				SBIA	
				Grislies	
HighRes3dNet^a^	543 subjects (single training-testing- validation-split)	443 (training)	Brain	Deepmedic	No
		50 (testing)		3D UNet	
		50 (validation)		VNet	
HolisticNet^a^	274 subjects (single training-testing- validation-split)	219 (training)	Brain	None	No
		28 (testing)			
		28 (validation)			
DenseVNet^a^	90 subjects (9-fold cross-validation)	80 per fold for training	Abdominal	DEEDS+JLF	Yes
				VNet	
		10 per fold for testing (90 across all folds)		VoxResNet	
Adapted UNet	109 subjects (10-fold cross-validation)	98–99 per fold for training	2D Prostate	Fine-grained RNN	No
		10–11 per fold for testing (109 across all folds)			

a http://www.niftynet.io/.

### 2.2 Segmentation metrics based on hold-out data

For comparison of automatic segmentations with the labelled ground-truth segmentations, two commonly adopted segmentation metrics are used, the Dice similarity coefficient (DSC) and the symmetric boundary distance (BD), given by:

$$\text{DSC}=\frac{2\left|X\cap Y\right|}{\left(\left|X\right|+\left|Y\right|\right)}$$

and

$$\text{BD}=\frac{D\left(X,Y\right)+D\left(Y,X\right)}{2}$$

respectively, where *X* and *Y* are the automatically predicted binary segmentations and the manual ground-truth, respectively. The DSC is an overlap measure with a range of [0,1]. *D*(*X, Y*) denotes the average Euclidean distance from boundary pixels in *X* to the closest boundary pixel in *Y*. These two metrics are adopted to directly measure the network generalisation ability in segmenting regions of interest on unseen hold-out data, here, whole gland segmentation of MRIs. Both measures were calculated on the largest resampled images with a size of [112, 128, 64] and an isotropic voxel size of [1, 1, 1] mm/voxel. The details of the validation experiment and the ground-truth segmentations used in this study are described in Sections 2.4 and 3.

### 2.3 Gland volume errors and estimated target registration errors

As a potential clinical application of prostate MRI segmentation, relative gland volume errors (GVEs) were also calculated between the network-segmented prostate gland and the manual ground-truth segmentation in the validation experiments by counting the positive foreground voxels in the binary masks. GVE is based on the absolute difference between V(X) and V(Y) representing the volumes of the automatic and ground-truth segmentations, respectively:

$$\text{GVE}=\frac{\left|V\left(Y\right)-V\left(X\right)\right|}{V\left(Y\right)}\times 100$$

Although an alternative regression network directly predicting volumes is possible, the GVE results may be useful to demonstrate a non-end-to-end prediction performance in a clinical scenario where, for example, whole gland segmentation is required for other tasks such as localising tumours.

**Fig. 3:**
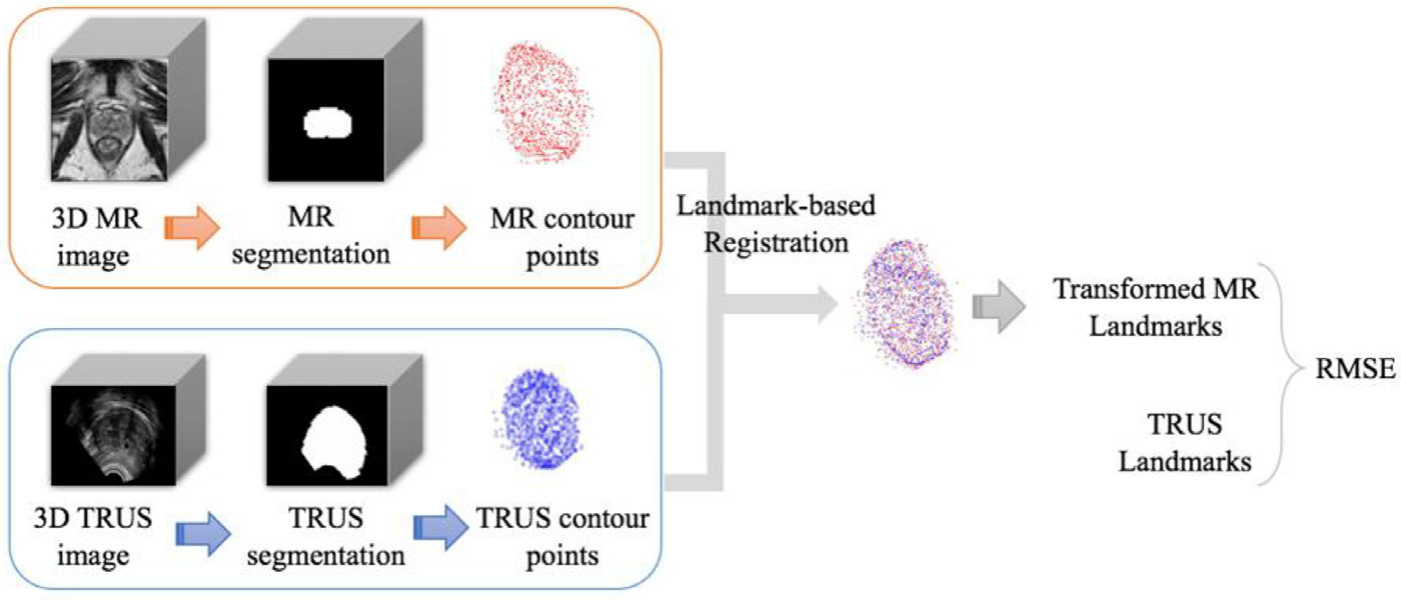
Segmentation-based registration pipeline.

The MRI-to-TRUS image registration can assist a range of TRUS-guided interventions, such as targeted biopsies and treatments (Ouzzane et al., 2015). Many proposed registration methods rely on matching prostate glands from (semi-) automated segmentation methods (Hu et al., 2012, Sparks et al., 2013, Hu et al., 2018, Meng et al., 2016, Oberlin et al., 2016, Kongnyuy et al., 2016, Zettinig et al., 2015). For the purposes of comparison and to ensure reproducibility, we adopt an open-source landmark-guided coherent point drift (LGCPD) algorithm (Hu et al., 2010)^1^ for deformable registration between the two point-sets representing the surfaces of the prostate gland segmentations from MRI and TRUS images. The latter segmentations are obtained from our previous work (Ghavami et al., 2018a) and remained fixed during all experiments for comparing different MRI segmentations. The apex and base points are identified for all the cases, used as guiding landmark pairs with known correspondence in the LGCPD algorithm. The registration produces a non-rigid transformation between the MRI and TRUS and this transformation is used to propagate MRI landmarks to the space of the TRUS landmarks. Once registered, the root-mean-square (RMS) distance between the transformed MR landmarks and TRUS landmarks is computed for each case as target registration errors (TRE), for different MRI segmentations reported in this study. The landmarks used included whole gland segmentations, urethra, visible lesions, junctions between the gland, gland zonal separations, vas deference, seminal vesicles, visible lesions, and other patient-specific point landmarks such as calcifications and fluid-filled cysts. A schematic of the registration workflow is displayed in Fig. 3.

### 2.4 Experiment design for network comparison

In real-world applications, network hyperparameters are optimised before further clinical testing and adoption. To facilitate a comparison that is informative to clinical practice, it is desirable to find the optimum hyperparameter configurations prior to comparing these six networks described in Section 2.1. It is also important to note that estimating segmentation performance directly from a hyperparameter optimisation procedure, e.g. estimated DSCs from a cross-validation, is subject to overfitting, which can introduce bias towards the entire data set used for the hyperparameter-optimising cross-validation. Therefore, we separated the data into development and hold-out sets. The development set is used for hyperparameter searching, whereas the hold-out set is used to report independent results on a dataset completely unseen during the network development (including searching for hyperparameter values).

**Fig. 4:**
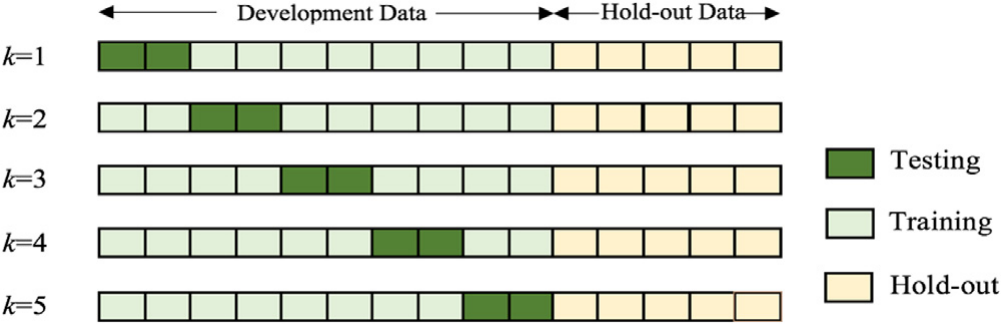
*k*-fold cross-validation example.

We adopted an exhaustive grid-search for tuning hyperparameters based on cross-validation (referred to as hyperparameter searching). First, each of the tested hyperparameters is sampled at a uniform interval from a respective pre-defined range; Second, each permutation of these sampled hyperparameters (hereafter referred to as “hyperparameter configuration”) is tested in a k-fold cross-validation experiment (here, *k* = 5). The details of the tested hyperparameter configurations are described in Section 3.3; Third, among these hyperparameter configurations, segmentation performance is evaluated by averaging the DSCs obtained from the k-fold network-training in the cross-validation; Finally, for each of the six network architectures, the hyperparameter configuration with the highest average DSC is selected. The division of the data used in this procedure is outlined in Fig. 4, and the data used in this study is described in Section 3.

The networks with the respectively-optimised hyperparameters are then tested on the hold-out data, for the purposes of comparison. All the segmentation (DSCs and BDs) and clinical measures (GVEs and TREs) described in Sections 2.2 and 2.3 were computed across all patients in the hold-out set. Both the two clinical measures (GVEs and TREs) and the segmentation measures (DSCs and BDs) are compared using a one-way analysis-of-variance (ANOVA) test at significance level of 0.05, among those produced by different networks. The ANOVA was followed by a multiple comparison, pairwise *t*-test of each pair of networks to see where the significance in the group means lies, if any significance is obtained using the ANOVA test. This multi-group testing procedure was also performed using non-parametric tests, i.e. using a Kruskal–Wallis (KW) test to test multiple group distributions.

## 3 Experiments

### 3.1 Imaging data and ground-truth segmentations

The complete data used for this work consisted of T2-weighted prostate MRIs taken from three different studies, SmartTarget Biopsy Trial (Hamid et al., 2018), INDEX Trial (Dickinson et al., 2013) and the PICTURE Trial (Simmons et al., 2013). 232 MRI volumes were available from the same number of patients. These trials share the same imaging protocols. Original image size and voxel size range from [256, 256, 25] to [512, 512, 30] and [0.35, 0.35, 3] to [0.86, 0.86, 3.6], respectively. All images were scanned using either a 1.5T or 3T Avanto™ Siemens scanner. Intensity values were normalised to zero-mean and unit-variance intensities for individual volumes.

For all 232 MRI volumes, manual segmentation of the prostate capsule boundary in consecutive transverse slices of each MRI volume was carried out by an expert clinical observer (either a radiologist or a urologist specialised in MRI-targeted procedures, verified by a senior radiologist). These segmentation labels provided the ground-truth for segmentation in both training and testing (development data) and validation (hold-out data), in this study.

Among the 232 image and segmentation data, 59 patient data from those taken from the SmartTarget Biopsy Trial (Hamid et al., 2018) were used as the hold-out data set and were not used during the hyperparameter searching. These patients had TRUS images available for further testing the subsequent MRI-TRUS image registration application in addition to the volume estimation results.

### 3.2 Implementation and network training

From the networks described in Section 2.1, for four of these; VNet, DenseVNet, HighRes3dNet and HolisticNet, the source code from NiftyNet (Gibson et al., 2017b) were directly used, while we implemented the published UNet (Cicek et al., 2016) and Adapted UNet (Ghavami et al., 2018a) in TensorFlow™ (Abadi et al., 2016) which is also made publicly available. Each network was trained with a 12GB NVIDIA^Ⓡ^ Pascal™ TITAN Xp general-purpose graphic process unit (GPU) on a high-performance computing cluster. The networks were run for 15,000 iterations. During each 5-fold cross-validation for hyperparameter searching, the remaining 173 patients were split into five folds, each containing 33–35 (∼20%) patient data. Given a hyperparameter configuration, each of these five folds was left out for testing, with the network trained using the other 138–140 (∼80%) training data. This was then repeated until every patient data was tested once, as shown in Fig. 4. This cross-validation procedure was repeated for each hyperparameter configuration (described in Section 3.3). Once the optimum hyperparameters were determined, five segmentations were predicted using the networks trained in the cross-validation on each of the 59 hold-out data. These five segmentations were then combined to generate the final segmentation using majority voting at each voxel, from which the segmentation accuracy and clinical metrics (described in Sections 2.2 and 2.3, respectively) were computed.

### 3.3 Hyperparameter configurations

To enable a computationally-feasible architecture comparison, four hyperparameters were varied to find the optimum combination of them for each network in this study, including input image size (after resampling from the original MRIs), initial learning rate of the Adam optimiser, regularisation weight of *L*^2^-norm on network parameters (weight decay) and number of initial feature channels. Table 2 summarises the four hyperparameters tested in this study, each with four different configurations, leading to a total of 256 hyperparameter configurations for each network.

**Table 2:** Different hyperparameter configurations used for the hyperparameters tuning of the different networks.

Training Hyperparameter	Value. 1	Value. 2	Value. 3	Value. 4
Input image size	[112, 128, 64]	[80, 96, 48]	[48, 64, 32]	[32, 48, 16]
Initial learning rate	10^−2^	10^−3^	10^−4^	10^−5^
Weight decay	0	10^−2^	10^−4^	10^−6^
Number of initial channels	4	8	16	32

The detailed values for these configurations are summarised in Table 2. The input images were resampled, from the centres of the image volumes, with respect to four different isotropic voxel sizes, [1, 1, 1] mm/voxel, [1.5, 1.5, 1.5] mm/voxel, [2, 2, 2] mm/voxel and [2.5, 2.5, 2.5] mm/voxel, with an empirically-set field of view. This resulted in the four sets of image sizes shown in the first row of Table 2. The field-of-view was cropped to reduce the computational burden using an estimate of a fixed physical region that is large enough to contain the entire prostate gland and most of the surrounding anatomical structures. The same field-of-view was used for all datasets in this study. The number of initial feature channels represents a measure of network size (Bonmati et al., 2018) and, together with input image size, are constrained by GPU memory. Although the minibatch size could also affect the network training (Radiuk, 2017, Smith et al., 2017), this was found to be relatively insignificant in our initial experiment. In this study, minibatch sizes, 2, 4, 8 and 16 were fixed according to four decreasing input image sizes, in order to maximise the usage of the GPU memory.

The other hyperparameters for each model architecture is kept the same as in the original publications. For the brevity of this paper, the reader is referred to the respective original publications and open-source code.

## 4 Results

### 4.1 Hyperparameter searching

Two-hundred and fifty-six different hyperparameter configurations were tested for the UNet and Adapted UNet, with the 5-fold cross-validation. The initial number of feature maps was not relevant for the other four networks, which had fixed model architectures without considering the change in the number of feature maps. Therefore, 64 hyperparameter configurations were tested for the VNet, HighRes3dNet, HolisticNet and DenseVNet. Based on the highest DSC values obtained from these experiments, the hyperparameter configurations found for each network is listed in Table 3. The networks trained with these hyperparameter configurations were used for the subsequent comparison reported here. The highest DSC values in addition to the 10th, 50th and 90th percentiles of the obtained DSC values from these experiments are also provided in Table 3.

**Table 3:** The selected hyperparameter configurations for each of the segmentation networks.

Network	Input image size	Initial learning rate	Weight decay	Number of initial channels	3D DSC max [10th,50th,90th] percentile
VNet	[32, 48, 16]	10^−4^	10^−4^	n/a	0.87 [0.84, 0.85, 0.87]
HighRes3dNet	[32, 48, 16]	10^−2^	0	n/a	0.87 [0.73, 0.84, 0.87]
HollisticNet	[32, 48,16]	10^−2^	10^−6^	n/a	0.87 [0.19, 0.68, 0.87]
DenseVNet	[32, 48, 16]	10^−3^	0	n/a	0.85 [0.76, 0.82, 0.85]
UNet	[48, 64, 32]	10^−2^	10^−6^	8	0.89 [0.67, 0.85, 0.88]
Adapted UNet	[48, 64, 32]	10^−3^	10^−6^	32	0.89 [0.67, 0.85, 0.88]

### 4.2 Segmentation accuracy

**Fig. 5:**
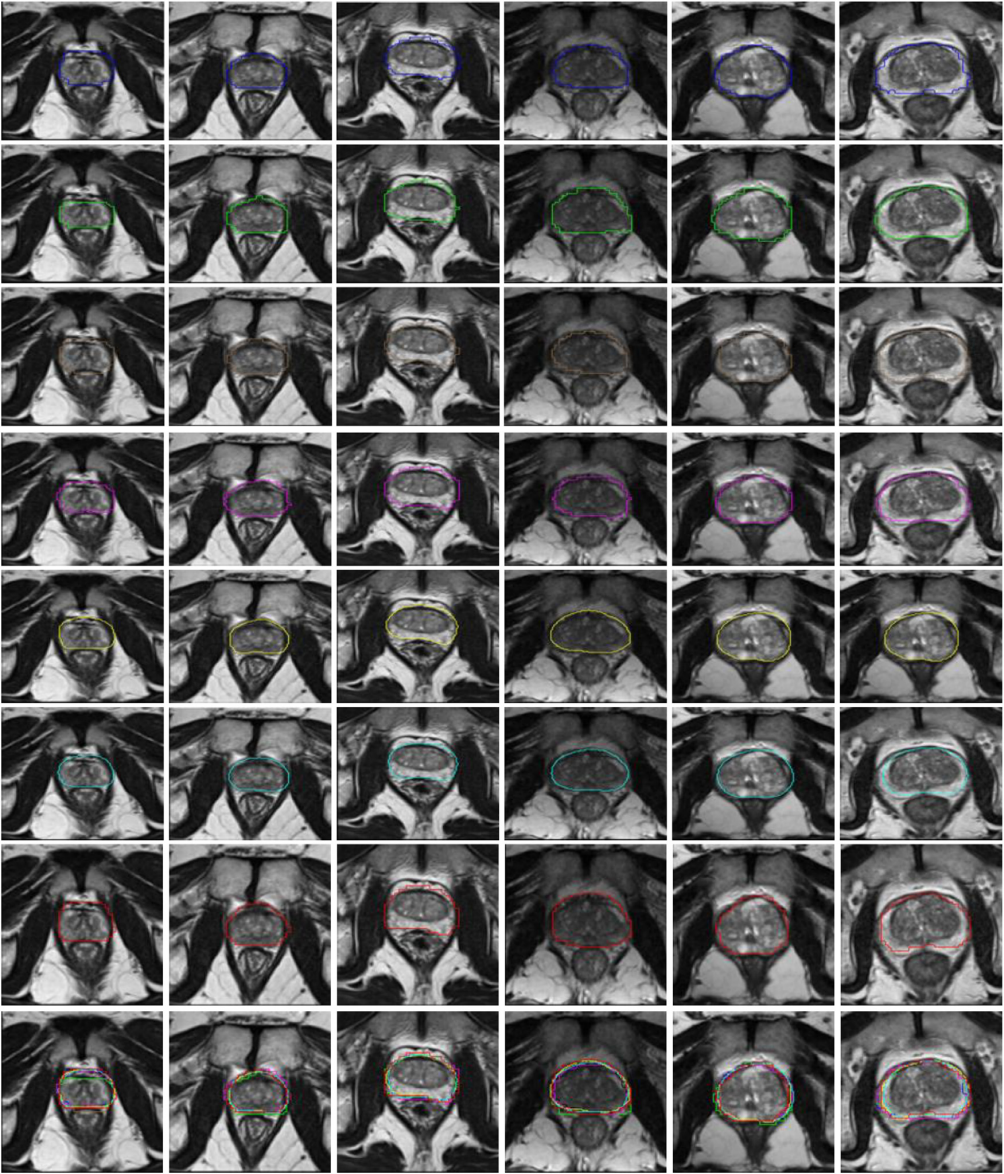
Automatic segmented prostate boundaries generated by different CNNs for 6 patients. The columns correspond to different patients and the rows correspond to different networks, with the last row showing the overlay of all networks. The first two columns are patients with DSC closest to the 25th percentiles, middle two columns are patients with DSC closest to 50th percentiles and the following two columns are patients with DSCs closest to the 75th percentiles. Blue shows the segmentation from HighRes3dNet, green from HolisticNet, brown from VNet, magenta the segmentation from DenseVNet, yellow the segmentation from the adapted UNet, cyan from UNet and red the manual segmentation.

**Fig. 6:**
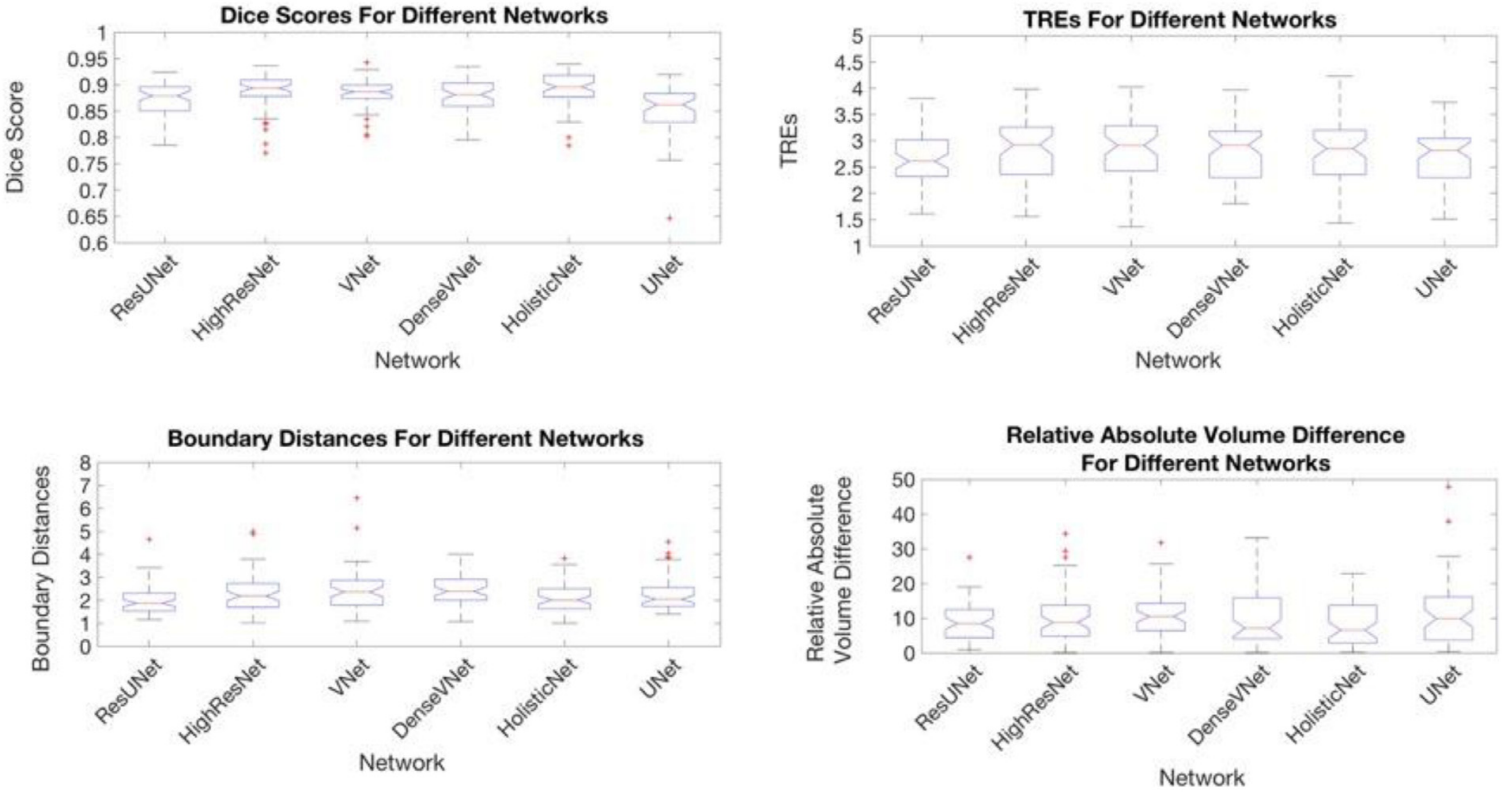
Box and Whisker plots of different measurement metrics for each of the segmentation networks.

Fig. 5 shows a comparison of example images overlaid with typical segmented prostate boundaries generated automatically from the trained networks, to illustrate qualitatively different levels of segmentation performance at 25th, 50th and 75th percentiles of DSC. The DSC and BD values are summarised in Table 4 and Fig. 6. The range of the median DSC was between 0.86 and 0.90, and the median BD ranged between 1.9 mm and 2.4 mm across the six networks. However, the one-way ANOVA test shows a statistically significant difference between the DSC, but not for the BD, with a *p-*value of 0.005 and 0.32, respectively, whereas the non-parametric Kruskal-Wallis test shows a statistically significant difference between both the DSC and the BD with p-value < 0.001 in both cases. The subsequent multiple comparison, based on Tukey's honest significance test, shows that, for the DSC, the difference was caused by the UNet, which produced, for example, *p-*values of 0.01, 4  ×  10^−3^ and 0.03, compared with VNet, HighRes3dNet and HolisticNet, respectively. No statistically significant difference was found between the other networks (*p*-values ranged from 0.84 to 1.00). The detailed pairwise multiple comparison results are also summarised in Table 5.

**Table 4:** Segmentation performance metrics, prostate volume calculations and target registration errors between the manual and automatic segmentation for each network.

Network	3D DSC mean ± std [25th,50th,75th] percentiles	Boundary distance (mm) mean ± std [25th,50th,75th] percentiles	Relative GVE difference (%) mean ± std [25th,50th,75th] percentiles	Target registration Error (mm) mean ± std [25th,50th,75th] percentiles	Number of parameters
UNet	0.84 ± 0.07	2.52 ± 1.48	11.29 ± 9.62	2.72 ± 0.51	294 k
	[0.83,0.86,0.88]	[1.73,2.07,2.57]	[3.65,9.83,16.03]	[2.30,2.82,3.05]	
VNet	0.88 ± 0.03	2.45 ± 0.91	10.71 ± 6.42	2.84 ± 0.59	71,044 k
	[0.87,0.89,0.90]	[1.78,2.36,2.88]	[6.32,10.44,14.25]	[2.43,2.91,3.18]	
HighRes3dNet	0.89 ± 0.03	2.33 ± 0.81	10.15 ± 7.54	2.86 ± 0.58	809 k
	[0.88,0.89,0.91]	[1.71,2.21,2.73]	[4.77,8.70,13.66]	[2.36,2.92,3.26]	
HolisticNet	0.88 ± 0.12	2.56 ± 3.22	9.60 ± 13.49	2.98 ± 1.25	4241 k
	[0.88,0.90,0.92]	[1.62,2.04,2.50]	[2.77,6.51,13.66]	[2.36,2.85,3.20]	
Dense VNet	0.88 ± 0.03	2.47 ± 0.66	10.78 ± 8.65	2.83 ± 0.57	867 k
	[0.86,0.88,0.90]	[2.00,2.37,2.92]	[4.04,7.06,15.80]	[2.30,2.91,3.18]	
Adapted UNet	0.87 ± 0.03	1.96 ± 0.61	8.99 ± 5.61	2.66 ± 0.45	9401 k
	[0.85,0.88,0.90]	[1.52,1.86,2.22]	[4.33,8.40,12.44]	[2.33,2.61,3.02]

**Table 5:** *p*-values between the different networks for the DSCs, BDs, GVEs and TREs.

Networks	DSC *p-value* (tukey-kramer)	BD *p-value* (tukey-kramer)	GVEs *p-value* (tukey-kramer)	TRE *p-value* (tukey -kramer)
UNet vs VNet	0.01	1.00	0.96	0.95
UNet vs HighRes3dNet	3.97e−3	0.99	0.83	0.92
UNet vs HolisticNet	0.03	1.00	0.29	0.42
UNet vs Dense VNet	0.03	1.00	0.43	0.97
UNet vs Adapted UNet	0.15	0.38	0.64	1.00
VNet vs HighRes3dNet	1.00	1.00	1.00	1.00
VNet vs HolisticNet	1.00	1.00	0.83	0.92
VNet vs Dense VNet	1.00	1.00	0.93	1.00
VNet vs Adapted UNet	0.96	0.54	0.99	0.77
HighRes3dNet vs HolisticNet	0.99	0.97	0.95	0.95
HighRes3dNet vs Dense VNet	1.00	1.00	0.99	1.00
HighRes3dNet vs Adapted UNet	0.84	0.79	1.00	0.71
HolisticNet vs Dense VNet	1.00	1.00	1.00	0.89
HolisticNet vs Adapted UNet	0.99	0.30	1.00	0.19
Dense VNet vs Adapted UNet	0.99	0.48	1.00	0.83

**Fig. 7:**
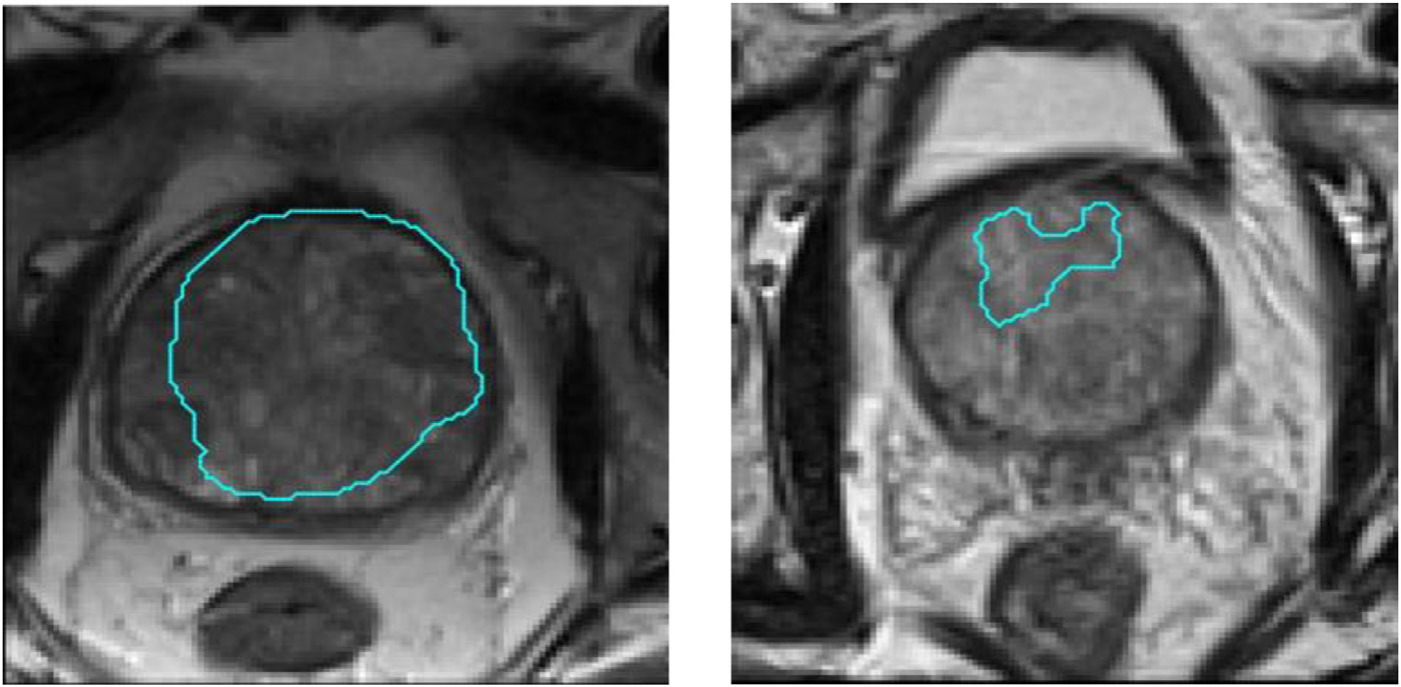
Overlay images of the automatic boundary segmentations from the UNet on top of the original prostate image for two outlier patients producing DSCs of smaller than 0.65.

Further investigations on the seemingly underperforming UNet revealed two outlier cases that produced DSC values lower than 0.65. Example slices for these cases are shown in Fig. 7. As reported in Section 4.1 and Table 3, a median DSC of 0.89 was obtained from the UNet training, which was not inferior to training errors from other networks, and indicates a clear example of parameter overfitting. A further discussion of the effect from these outliers on the subsequent clinical tasks are discussed in Section 4.3.

### 4.3 Volume estimate errors and target registration errors

Using the segmentations reported in the previous section, the relative GVE and TRE values are also summarised in Table 4 and Fig. 6. These networks estimated the gland volumes with a median relative GVE between 6.5% and 10.4%, and the median TREs were lower than 3 mm. Most interestingly, no statistically significant difference was found among these networks using the one-way ANOVA test, either in GVE (*p*-value = 0.34) or in TREs (*p*-value = 0.26). This lack of significance was also confirmed by the non-parametric Kruskal–Wallis test, with a *p*-value of 0.60 and 0.39 for the GVE and TRE, respectively. Additional pairwise multiple comparison results are summarised in Table 5.

**Fig. 8:**
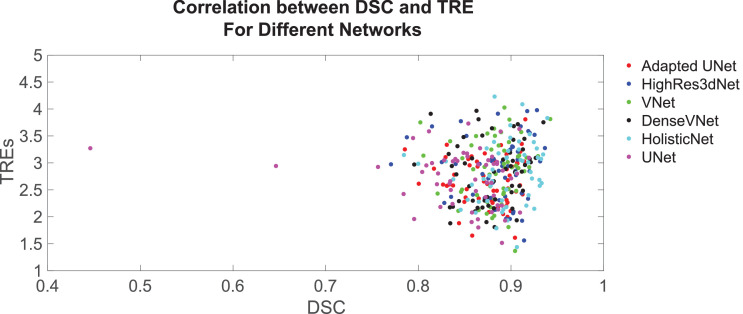
Plot showing relationship between DSCs and TREs for patients across all six networks, represented by the six different coloured points.

A subject-level comparison of the segmentation accuracy, measured by the DSC, and the corresponding registration accuracy, measured by the TRE, is illustrated in Fig. 8. The results show little visual correlation between these two measures in any tested networks; a Pearson's correlation coefficient of 0.015 was obtained between the DSC and TRE. The two outlier cases with the UNet (as reported in Section 4.2), which were predominantly responsible for the significant difference in segmentation performance, did not reduce the registration accuracy, with corresponding TREs of 2.94 mm and 3.27 mm. In these cases, the adverse effect from the relatively poor segmentation was probably mitigated by multiple landmarks and deformation regularisation used in the registration algorithm, demonstrating that, in the current settings, a difference between DSC values would not make a difference in clinical use of the segmentation such as MRI-to-TRUS registration tested in this study.

## 5 Discussion and conclusion

In this study, six recently-proposed CNNs were compared to segment prostate glands in MRIs. The segmentation performance in terms of the DSC region overlap measure and the BD was quantified for 232 patient datasets with expert labels provided by experienced clinicians. Although the original purpose of our work was not to recommend any particular network architecture over any other, an extensive comparison including 3840 trained models was carried out to ensure a practically-feasible and fair comparison of network generalisation. The results are reported from a hold-out dataset, after completing parameter searching based on cross-validation. Furthermore, two real clinical tasks were tested in which the automatically-predicted segmentations were used for prostate volume estimation and multimodal image registration. The results, in terms of the relative GVE and TRE, were also compared statistically among all the networks. We believe that this it is the first time that a comparison experiment based on a single data set of this size has been reported for MRI-TRUS prostate segmentation. It is also the first study to investigate how errors in MRI prostate segmentation influence the accuracy of clinical workflow tasks where the segmented boundaries are input data. One such task is the estimation of the prostate volume. As briefly mentioned in Section 1, this measure is important for investigating the effect of a drug therapy on the prostate over time. For example, Moore et al. (2017) showed a 15% reduction in prostate volume and 34% reduction in tumour volume in patients given dutasteride, a drug for treating prostatic hyperplasia. From the results of our experiments, the median difference in GVE of between 6.5% and 10.4% using segmentations produced automatically by different networks (see Table 4) would be significant in this application. Furthermore, as our results suggest, the GVE would not necessarily be reduced by selecting another network tested in this study that segments the prostate boundary more accurately.

For the MRI-to-TRUS registration application, all of the networks tested resulted in a median TRE between 2.6 mm and 2.9 mm, which is comparable to other segmentation-based registration methods in the literature (Narayanan et al., 2009, Hu et al., 2012, Zettinig et al., 2015). As shown by Van de Ven et al. (2013), a TRE of 3.1 mm or less is required to detect a clinically significant tumour volume. From the results presented in Table 4, the percentage of patients with a TRE smaller than 3.1 mm was 80%, 64%, 71%, 67%, 73% and 82% for UNet, VNet, HighRes3dNet, HolisticNet, Dense VNet and Adapted UNet respectively.

Moreover, we believe that the results from this work may corroborate the findings of a number of previous studies in which caution has been raised over the interpretation of the value of some segmentation metrics, and the resulting league table positions in segmentation challenges (Gibson et al., 2017a, Reinke et al., 2018). With evidence from the prostate segmentation in MRIs, we found that a statistically significant difference in the DSC between segmentations produced by two CNNs, does not necessarily lead to any detectable impact in other computational tasks within a clinical workflow that use these segmentations. As shown in Table 5, unlike in the DSC results, no statistically significant difference in BDs was found between the networks in this work using the ANOVA. This itself raises interesting questions for further comparison such as, “Does BDs correlate with GVEs more than it does with DSCs?” or “Is BD a better predictor of TRE than DSC is?”.

Our conclusions need to be considered with limitations such as data size, access to segmentation networks that are designed for these clinical applications and the choice of method using these segmentations. For instance, the registration algorithm used in this work is an open-source algorithm that produced acceptable registration results, but it may be interesting to compare with other methods with or without using segmentations. Also, in this work, we have focused on the performance of the segmentation networks in accuracy. Other aspects of the networks which could potentially also influence the clinical adoption, such as training- and inference time, have not yet been optimised and compared.

With the increasing use of deep learning in medical imaging, especially with different networks which are proposed to be used in clinical practice, representing prediction uncertainty is of importance. The uncertainty can arise from noisy data, the sampling of training data, and uncertainty in the model parameters and the network structure (Gal, 2016). Although outside the current scope of this paper, it would be interesting to investigate and compare the impact of network uncertainties both on segmentation accuracy and subsequent clinical metrics in future research.

Established segmentation error metrics, such as the Dice Score, are useful for formulating loss functions for training learning-based algorithms, and for evaluating and comparing the segmentation accuracy of different network architectures. Therefore, the development of new architectures that are demonstrated to be more accurate using such measures remains a well-justified and important engineering goal. However, by reporting the quantitative results in real clinical applications investigated in this work, we hope it will influence the scope of future research and development to consider carefully the accuracy of specific downstream tasks **of interest** within a computational pipeline for the specific clinical application of interest. This work serves as a starting point for this shift by demonstrating that any found statistical significance cannot be generalised to downstream clinical tasks without further validation.

## Declaration of Competing Interest

The authors declare that they have no known competing financial interestsor personal relationships that could have appeared to influence the work reported in this paper.

## Footnotes

1 https://github.com/YipengHu/matlab-common-tools/tree/master/GuidedCPD.
